# Whispers in the genome: The hidden grammar of tomato fruit development

**DOI:** 10.1093/plcell/koaf248

**Published:** 2025-10-15

**Authors:** Andrea Gómez-Felipe

**Affiliations:** Assistant Features Editor, The Plant Cell, American Society of Plant Biologists; Department of Biology, Indiana University, Bloomington, IN 47405, USA

Enhancers are regulatory DNA elements that control when, where, and how many genes are expressed. They can act in *cis* or *trans*, occur in noncoding and coding regions, and may produce enhancer RNAs that contribute to their function (Inoue and Ahituv 2015). While enhancer activity has been studied in various systems, their roles in plant development, particularly in fruits, remain unclear.

Fruit-specific gene expression is critical for proper development, ripening, and metabolic regulation, but the regulatory elements and sequence features that control this process remain largely unexplored. To address this gap, Yaxin Deng, Weihua Zhao, and colleagues (Deng et al. 2025) systematically mapped and characterized enhancers driving fruit-specific expression in tomato using a massively parallel reporter assay (MPRA). They synthesized 11,180 promoter fragments from 1,118 fruit-specific genes and linked each fragment to a luciferase reporter gene with unique DNA barcodes, enabling high-throughput measurement of enhancer activity. The MPRA library was transiently expressed in tomato fruits at 3 developmental stages—mature green, breaker, and red ripening—as well as in leaves to assess tissue specificity (Fig.).

**Figure.**
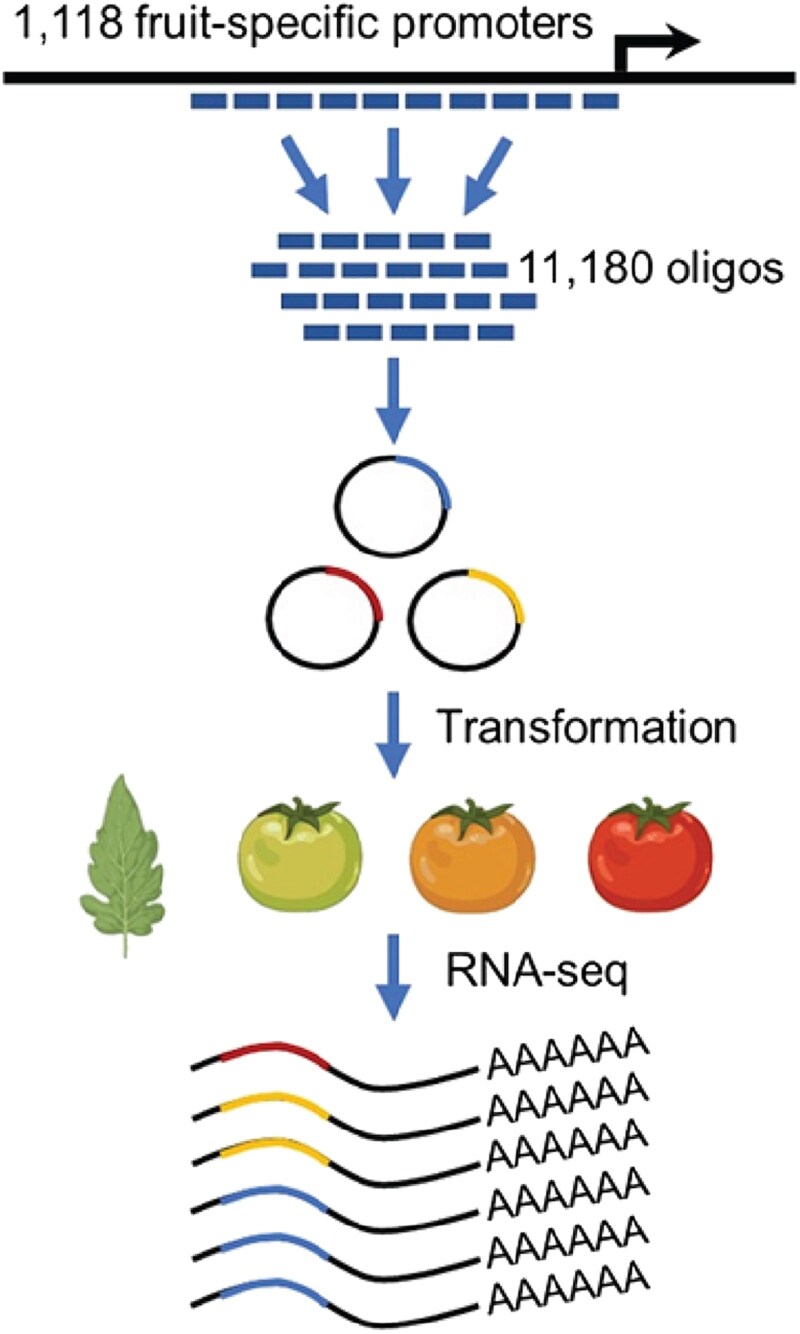
Experimental overview of the MPRA. A library of 11,180 synthesized 160–base pair promoter fragments was cloned upstream of a reporter gene and transiently expressed in tomato fruits via *Agrobacterium* infiltration. Fruit specificity was assessed by parallel transformation in leaves, and enhancer activity was quantified by sequencing DNA input and RNA barcodes. Adapted from Figure 1 of Deng et al. (2025).

Analysis of the resulting data revealed 2,436 functional enhancers, including a subset that remained highly active across developmental stages. The findings showed that intrinsic enhancer activity is largely independent of the position relative to transcription start sites. Many of these enhancers overlap with chromatin-accessible regions and regulate genes involved in fruit ripening, stress responses, and metabolic processes, underscoring their critical roles in fruit development.

To uncover the regulatory code underlying enhancer activity, the authors trained a convolutional neural network on the MPRA data to predict enhancer activity from DNA sequences. The model accurately distinguished fruit from leaf enhancers and identified motifs predictive of fruit-specific activity. Further analysis revealed that conserved patterns were concentrated in flanking regions of enhancers, and even subtle motif differences could influence tissue specificity. Experimental validation confirmed the functional role of these motifs in fruit-specific expression.

Building on this framework, the authors used iterative in silico evolution to design synthetic enhancers. Starting from 160–base pair DNA sequences, they applied model-guided mutagenesis to progressively enhance predicted fruit-specific activity. After multiple iterations, enhancer activity and tissue specificity increased substantially. To test their functionality in vivo, 6 candidate sequences were selected for reporter assays, and 5 of them drove strong fruit-specific expression, with motif enrichment and subtle architectural changes underlying their activity.

Overall, this study provides a comprehensive map of fruit enhancers, deciphers the sequence features underlying their activity, and shows how deep learning can guide the rational design of synthetic regulatory elements. By integrating high-throughput assays, computational modeling, and synthetic biology, it establishes a framework for understanding tissue-specific regulation and offers tools for engineering synthetic enhancers to precisely control gene expression in plants. These findings have broad implications for developmental biology, crop improvement, and the design of tailored regulatory circuits in plant biotechnology.

## Recent related article in *The Plant Cell*

- Akagi et al. (2022) employed a convolutional neural network model to predict gene expression patterns in tomato fruit, based on *cis*-regulatory elements (CREs) in regulatory regions.

## Data Availability

N/A

## Dive Curated Terms

The following phenotypic, genotypic, and functional terms are of significance to the work described in this paper:

fruit AmiGo: PO:0009001
